# Sex-Specific Disparities in Outcomes of Transcatheter Edge-to-Edge Repair for Mitral Regurgitation: A Multicenter “Real-World” Analysis

**DOI:** 10.3390/jcm12237231

**Published:** 2023-11-22

**Authors:** Felix Ausbuettel, Sebastian Barth, Georgios Chatzis, Kiarash Sassani, Dieter Fischer, Sebastian Weyand, Julian Mueller, Harald Schuett, Bernhard Schieffer, Ulrich Luesebrink, Christian Waechter

**Affiliations:** 1 Department of Cardiology, University Hospital Marburg, Philipps University Marburg, Baldingerstraße, 35043 Marburg, Germany; georgios.chatzis@uk-gm.de (G.C.); kiarash.sassani@gmail.com (K.S.); julianmueller240491@gmail.com (J.M.); harald.schuett@uk-gm.de (H.S.); bernhard.schieffer@staff.uni-marburg.de (B.S.); ulrich.luesebrink@staff.uni-marburg.de (U.L.); 2 Department of Cardiology, Cardiovascular Center Bad Neustadt/Saale, Von-Guttenberg-Straße 11, 97616 Bad Neustadt/Saale, Germany; sebastian.barth@campus-nes.de; 3 Department of Cardiology, Cardiovascular Center Rotenburg/Fulda, Heinz-Meise-Straße 100, 36199 Rotenburg/Fulda, Germany; dieterfischer@yahoo.de; 4 Department of Cardiology, Ostalb Clinic Aalen, Im Kaelblesrein 1, 73430 Aalen, Germany; sebastian.weyand@kliniken-ostalb.de

**Keywords:** gender, MitraClip, PASCAL, percutaneous mitral valve repair, atrial fibrillation, outcome, arrhythmia

## Abstract

Background: mitral regurgitation (mr) is the most common valvular heart disease (vhd) in the elderly and tends to be more prevalent in women. while relevant sex differences in outcomes are evident in surgically treated collectives, there are very limited and conflicting sex-specific data for the growing cohort of patients undergoing transcatheter edge-to-edge repair (teer). Objective: to investigate whether sex impacts procedural safety and efficacy, and in-hospital- and long-term outcomes, after teer for mr. Methods: in a multicenter observational cohort study, patients who underwent teer were stratified by sex and relevant outcome measures, and analyzed using multivariable cox regression and propensity score matching (psm). Results: a total of 821 patients were analyzed, of whom 37.4% (307/821) were female. compared to male patients, females were significantly older (77 ± 8.5 vs. 80.4 ± 6.7 years, *p* = 0.03), and had less coronary artery disease (cad, 67.7% vs. 53.1%, *p* < 0.0001) and a higher proportion of preserved left ventricular function (lvef > 50%, 32.5% vs. 50.5%, *p* > 0.0001). safety and efficacy of the teer procedure and in-hospital mortality did not differ between the sexes. after psm, women showed significantly better survival 3 years after teer compared to men (60.7% vs. 54.2%, *p* = 0.04) and a lower risk of all-cause death according to multiple cox regression (hr 0.8, 95% ci 0.6–0.9, *p* = 0.02). after sex-specific stratification for concomitant atrial fibrillation (af), the most common comorbidity in the present collective, women with af experience significantly worse adjusted survival compared to women without af (53.9% vs. 75.1%, *p* = 0.042) three years after teer and lose the survival advantage over men. Conclusions: female patients are older and less comorbid than males undergoing TEER. The TEER procedure is equally safe and effective in both sexes. While in-hospital mortality did not differ, female patients experienced a significantly better adjusted long-term survival compared to male patients. Concomitant AF offsets the prognostic advantage of females over males and, in contrast to males, significantly impairs long-term survival in women undergoing TEER. Further research is warranted to elucidate underlying causes for the observed sex disparities and to develop sex-tailored treatment recommendations.

## 1. Introduction

Valvular heart disease (VHD) is a major contributor to reduced physical capacity, loss of quality of life and early mortality, and poses a major health concern worldwide [1]. In high-income countries, functional and degenerative forms of VHD are pervasive, with the vast majority of cases being diagnosed in the population over 65 years of age [2]. In this regard, men and women are equally impacted by VHD, albeit relevant sex-specific differences exist in the prevalence of the diseased valve and underlying pathogenesis [3,4,5]. While male patients are slightly more likely to be affected by aortic stenosis, the prevalence of mitral regurgitation (MR), which is the most common form of VHD in the population over 75 years of age, tends to be higher in female patients [1,3]. As the prevalence of MR is predicted to increase further in the coming decades due to improved survival rates and ageing societies, it can be expected that the number of women affected will also grow disproportionately [1,6].

In addition to the outlined differences in the epidemiology of MR, however, there appear to be even more profound sex-specific disparities in the subsequent management. Thus, women have been shown to be greatly underrepresented in clinical trials of both surgical and percutaneous MR therapies, to have lower access to surgery, and to be referred for intervention at a later stage of the disease [2,7]. While females undergoing MV surgery have higher perioperative mortality and lower long-term survival compared with male patients [8,9,10], it is unclear whether this also applies to the growing cohort of high-risk patients treated percutaneously. Only very limited and conflicting results are available on the impact of sex on clinically relevant endpoints in patients undergoing transcatheter edge-to-edge repair (TEER) of the mitral valve (MV). 

To address the need for precise sex-based recommendations and treatment strategies, the present study aims to investigate the disparities in short- and long-term outcomes between male and female patients undergoing TEER in a large, well-characterized, multicenter “real world” collective. This also includes a focus on the sex-specific prognostic impact of atrial fibrillation (AF), one of the most common and adverse comorbidities in this unique patient population.

## 2. Material and Methods

### 2.1. Data Collection and Definitions

Data on all consecutive patients in whom TEER was planned after a multidisciplinary heart team consensus decision at four tertiary cardiac centers in Germany between October 2011 and December 2022 were partially collected retrospectively in registries of the participating centers and subsequently pooled for the present analysis. Eligibility for TEER was defined according to the relevant guidelines [11]: In general, patients with severe symptomatic primary MR and those with secondary MR who remained severely symptomatic in spite of optimal guideline-directed heart failure management, who were at high or prohibitive risk for surgery, and who fulfilled the echocardiographic criteria for eligibility for TEER. The multidisciplinary heart teams were guided principally by the echocardiographic criteria defined in the Endovascular Valve Edge-to-Edge Repair Study (EVEREST) II for primary MR and in the Cardiovascular Outcomes Assessment of the MitraClip Percutaneous Therapy for Heart Failure Patients with Functional Mitral Regurgitation (COAPT) study for secondary MR [12,13]. Regarding procedural outcome, successful TEER was defined as MR reduction to less than or equal to moderate severity and a pressure gradient across the mitral valve of 5 mmHg or less after device implantation. The definitions of AF types and therapies, procedural aspects, and patient selection have previously been published [14,15]. In brief, paroxysmal AF was delineated as lasting seven days or less. All episodes of AF lasting longer than seven days were defined as non-paroxysmal AF, independently of the underlying AF treatment regimen being pursued. Major adverse cardiac and cerebrovascular events (MACCEs) and bleeding complications were defined and reported in accordance with the Mitral Valve Academy Research Consortium (MVARC) recommendations [16].

The study was conducted according to the Declaration of Helsinki guidelines and received approval from the Ethics Committee of the Philipps University of Marburg, Department of Medicine, Germany (reference number 120/18). Due to the nature of an observational study, patient consent was deemed unnecessary and waived after review and approval by the responsible ethics committee.

### 2.2. Statistical Analyses

All statistical analyses were carried out using R Studio V3.6.1 (R Foundation for Statistical Computing, Vienna, Austria), including the “survival”, “MatchIt”, “survminer”, “stddiff”, “My.Stepwise”, and “dplyr” packages, and GraphPad Prism 6.0 (Dotmatics, Boston, MA, USA). Continuous variables are reported with mean and standard deviation for normally distributed variables and with median and interquartile range (IQR: 25th–75th percentile) for non-normally distributed variables. Categorical variables are presented as frequencies and percentages (%). Differences between two groups were compared for categorical variables using the chi-square test when the expected cell size was ≥20 and Fisher’s exact test when the expected cell size in one or more cells was <20. For continuous variables, Student’s *t* test was used for normally distributed variables and Wilcoxon’s test was used for non-normally distributed variables. The normal distribution of continuous variables was validated using the Shapiro–Wilk test. A two-sided *p*-value of <0.05 was considered statistically significant. To account for differences in baseline characteristics and to achieve the most unbiased comparison possible between outcomes of male and female patients, a propensity score matching (PSM) analysis was performed using nearest neighbor matching with the difference in the logit of estimated propensity scores adjusted to 0.2 standardized range of measurement. To include as many subjects as possible from the total cohort in the analyses, the groups under study were matched in different ratios. The matching ratio was based on the group with the smallest number of patients, and on ensuring a sufficient balance of baseline characteristics. Statistically significantly different (*p*-value limit 0.05) parameters in the corresponding baseline characteristics and previously published and generally accepted mortality predictors, as well as mortality predictors determined or confirmed by univariable and multivariable Cox regression analyses in the present collective, were used to select the appropriate matching parameters. The selected matching parameters were age, coronary artery disease, left ventricular ejection fraction, pre-existing cardiac resynchronization therapy, chronic obstructive pulmonary disease, New York Heart Association (NYHA) functional class IV, angiotensin-receptor neprilysin inhibitor use, STS risk score, and concurrent severe tricuspid regurgitation. Before and after matching, a time-to-event analysis was performed for both sexes in general, as well as for both sexes, according to the status of concomitant AF using the Kaplan–Meier method; differences between groups were compared with the log-rank test. Both univariable and multivariable Cox regression were performed to determine independent predictors of mortality. Variables with *p* < 0.1 in the univariate analysis were included in the multivariable Cox regression model. The primary end point of the survival analyses was death from any cause.

### 2.3. Missing Data

In cases of insufficient follow-up data, they were augmented by a survival query to the responsible registry office for patients who were lost to follow-up. Despite all attempts, 55 patients (6.7%) could not be followed up because of an unreported residential change during the indicated study period. There was no evidence of informative missingness and no significant impact of “lost to follow-up” patients on the results presented. Supplemental Table S1 presents baseline characteristics of patients lost to follow-up in comparison with the overall cohort of patients studied.

## 3. Results

During the study period, 868 patients underwent TEER for severe MR at the participating heart centers. Due to insufficient mitigation of MR severity by the percutaneous approach, surgical treatment was required in 32 patients (3.7%), and 15 patients (1.7%) continued to receive conservative management and were therefore excluded from the further analyses. Sex had no effect on the success of the TEER procedure (Odds Ratio (OR) 0.96, 95% Confidence Interval (CI) 0.6–1.5, *p* = 0.9).

### 3.1. Baseline Characteristics

The patient cohort studied included a total of 821 patients, of whom 307 (37.4%) were of female and 514 (62.6%) of male sex. The baseline data for the total cohort and the data stratified by sex are presented in Table 1. Female patients were statistically significantly older, and had less coronary artery disease (CAD) and previous coronary artery bypass grafting (CABG), and a higher proportion of preserved left ventricular ejection fraction (LVEF); conversely, they also had a lower proportion of reduced LVEF, fewer pre-existing implantable cardioverter/defibrillator (ICD) and cardiac resynchronization therapy (CRT) devices, and, more frequently, mildly reduced left ventricular ejection fraction (LVEF) than male patients. Consistent with the latter finding, female patients were statistically significantly less likely to be treated with angiotensin-neprilysin and sodium-glucose transporter-2 inhibitors and were more often medicated with angiotensin-converting enzyme or angiotensin-1 inhibitors than male patients. Regarding the etiology of MR, also consistent with the differences described, the proportion of functional MR was lower in women than in men (47.9% vs. 55.4%, *p* = 0.1), although this was not statistically significant.

Regarding the procedural characteristics, the number of clips implanted in female patients was statistically significantly lower, and the duration of the procedure was consequently shorter than that in male patients, with no significant differences in MR reduction efficacy and length of hospital stay. Table 2 shows the procedural characteristics and efficacy outcomes for the total cohort and stratified for both sexes.

### 3.2. Complications and Short-Term Outcome

There were no statistically significant differences between the sexes in procedural complications or short-term outcomes that occurred during the index hospitalization. Table 3 reports procedure-related complications and rates of in-hospital cardiovascular and all-cause death.

### 3.3. Long-Term Outcome

**Figure 1:**
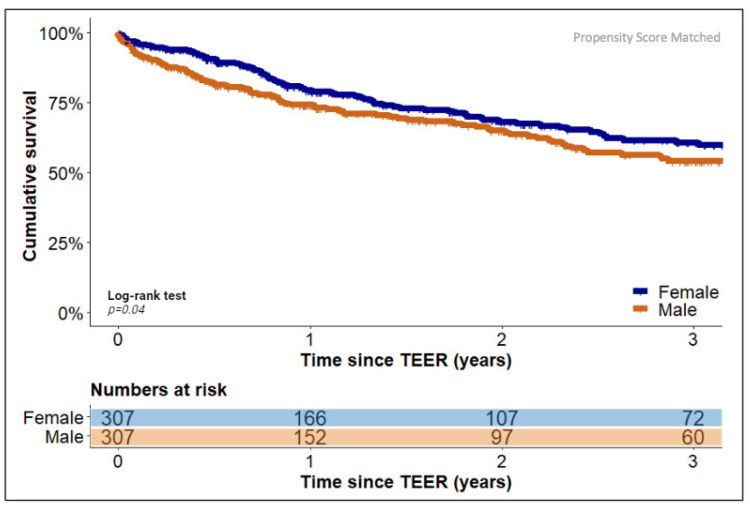
Estimated cumulative survival of TEER patients stratified by sex. Kaplan–Meier plot showing cumulative survival of female (blue graph) and male (orange graph) patients after propensity score matching. The graph indicates mean.

In an unadjusted Kaplan–Meier analysis, female patients were found to have a statistically significantly higher cumulative survival rate three years after the TEER procedure compared with male patients (60.7% [186/307] vs. 51.1% [263/513], *p* = 0.0085). However, to adjust this finding for the reported differences in baseline characteristics between the sexes, a propensity score matching (PSM) analysis was performed. This adequately balanced all relevant parameters (Table 1), and the subsequent Kaplan–Meier analysis confirmed the statistically significantly better cumulative survival rate after three years following the TEER procedure in female compared to male patients (60.7% [186/307] vs. 54.2% [166/307], *p* = 0.04). Figure 1 displays the Kaplan–Meier graph after PSM adjustment.

In addition, parameters statistically significantly related to long-term all-cause mortality (male sex, atrial fibrillation (AF), chronic obstructive pulmonary disease (COPD), CAD, high-grade tricuspid regurgitation (TR), pre-existing ICD, glomerular filtration rate (GFR), New York Heart Association (NYHA) functional class IV) were identified and included in a multiple Cox regression model. This model showed that in the cohort of TEER patients studied, male sex (Hazard Ratio (HR) 1.3, 95% CI 1.1–1.7, *p* = 0.02), concomitant high-grade TR (HR 1.8, 95% CI 1.4–2.4, *p* < 0.0001), COPD (HR 1.6, 95% CI 1.2–2.0, *p* < 0.001), GFR < 30 mL/min (HR 1.4, 95% CI 1.05–1.8, *p* = 0.02), and AF (HR 1.3, 95% CI 1.03–1.7, *p* = 0.046) were independently associated with a statistically significant higher, and, vice versa, female sex (HR 0.8, 95% CI 0.6–0.9, *p* = 0.02) with a statistically significant lower, risk of death from any cause after a median follow-up of 397 days (IQR 890 days).

### 3.4. Sex-Specific Effects of Concomitant Atrial Fibrillation on Long-Term Outcome

Considering that AF is the most common comorbidity after arterial hypertension in the present patient cohort, with an overall prevalence of 74.1%, and is independently associated with a higher risk of mortality, we aimed to investigate the sex-specific impact of concomitant atrial fibrillation on long-term outcomes. Thus, both male and female patients were stratified according to their history of concomitant AF, and Kaplan–Meier analyses were subsequently performed.

This showed that male patients with concomitant AF were statistically significantly older, less likely to have had prior CABG surgery, and more frequently presented with concomitant high-grade TR than males without a history of AF. With respect to medication, male subjects with concomitant AF were statistically significantly more often prescribed digitalis compared with male patients without AF. Table 4 (left column) displays the baseline characteristics of male patients stratified by AF status. 

**Figure 2:**
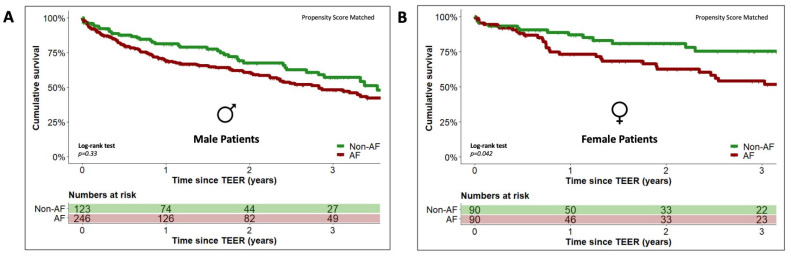
Estimated cumulative survival of male (**A**) and female (**B**) TEER patients stratified by status of concomitant atrial fibrillation. Kaplan–Meier plots showing cumulative survival of male (panel (**A**)) and female patients (panel (**B**)) with concomitant atrial fibrillation (AF, red graphs) and without a history of AF (green graphs) after propensity score matching. The graphs indicate means.

In an unadjusted Kaplan–Meier analysis there was no statistically significant difference in the cumulative survival at three years after TEER procedure between male patients with and without a history of concomitant AF (49.3% [193/391] vs. 57.1% [70/123], *p* = 0.4), which was confirmed by Kaplan–Meier analysis after adjustment for the recorded baseline differences (see Table S2) using PSM (48.2% [119/246] vs. 57.1% [70/123], *p* = 0.33). Figure 2 (panel A) depicts the graph of the corresponding Kaplan–Meier analysis after PSM adjustment. Cox regression analysis also identified no statistically significant association between concomitant AF and mortality in the male TEER patient cohort (HR 1.1, 95% CI 0.8–1.6, *p* = 0.4).

Stratification by AF status in the female TEER patient cohort revealed that females with a history of concomitant AF had a statistically significantly higher STS risk score, and more frequently exhibited concomitant arterial hypertension as well as high-grade TR, compared to female patients without AF. In addition, female TEER patients with concomitant AF were also more likely to be treated with digitalis than female patients without AF. Table 4 (right column) provides baseline characteristics of female TEER patients stratified by AF status. 

An unadjusted Kaplan–Meier analysis yielded a statistically significantly lower cumulative survival at three years after the TEER procedure for female patients with concomitant AF compared to female patients without a history of AF (55.4% [120/217] vs. 75.1% [68/90], *p* = 0.048). After adjustment for observed baseline differences using PSM (see Table S3), the subsequently performed Kaplan–Meier analysis confirmed this finding (53.9% [49/90] vs. 75.1% [68/90], *p* = 0.042). The graph of the corresponding Kaplan–Meier analysis after PSM adjustment is given in Figure 2B.

A multivariable Cox regression model including AF, NYHA functional class IV, secondary MR etiology, high-grade TR, LVEF < 30%, and GFR < 30 mL/min also identified concomitant AF to be independently associated with a higher mortality risk in the cohort of female TEER patients (HF 1.7, 95% CI 1.02–2.8, *p* = 0.04).

**Figure 3:**
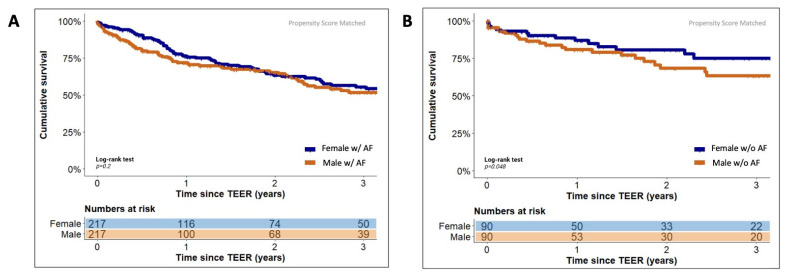
Estimated cumulative survival of male and female TEER patients with (**A**) and without (**B**) concomitant atrial fibrillation. Kaplan–Meier plots showing cumulative survival of male (orange graphs) and female patients (blue graphs) with concomitant atrial fibrillation (AF, panel (**A**)) and without a history of AF (panel (**B**)) after propensity score matching. The graphs indicate means.

Direct comparison of female and male TEER patients with concomitant AF showed no statistically significant differences in cumulative survival three years after the TEER procedure in both the unadjusted (55.4% [120/217] vs. 49.3% [193/391], *p* = 0.09) and in the PSM-adjusted (55.4% [120/217] vs. 51.6% [112/217], *p* = 0.2) Kaplan–Meier analysis. In contrast, female TEER patients without a history of AF exhibited a statistically significantly better three-year cumulative survival than male TEER patients without AF in the unadjusted (75.1% [68/90] vs. 57.1% [70/123], *p* = 0.01) as well as in the PSM-adjusted (75.1% [68/90] vs. 63.4% [57/90], *p* = 0.048) Kaplan–Meier analysis. The use of oral anticoagulants and the underlying therapy of concomitant AF did not differ statistically significantly between the sexes regarding the treatment regimen of rate or rhythm control and the corresponding therapeutic measures used for this patient group. Figure 3 illustrates the corresponding PSM-adjusted Kaplan–Meier plots for the comparison of female and male TEER patients with concomitant AF and without a history of AF, respectively. Baseline characteristics for these comparisons before and after PSM adjustment are shown in Tables S4 and S5.

## 4. Discussion

Despite decades of campaigns to increase awareness of the sex-specific impact of cardiovascular disease, sex-equitable health care remains a distant prospect. As a result, cardiovascular disease in women continues to be understudied, underdiagnosed, and undertreated [17]. Thus, we aim to contribute to the development of concise sex-specific recommendations and treatment strategies, and present an in-depth sex-specific analysis of the safety, efficacy, and short- and long-term outcomes of TEER in MR in a large, well-characterized, multicenter “real-world” population. In this regard, we firstly show that, compared with the prevalence of MR in the general population, female patients are also underrepresented in the present collective with a proportion of only 37.4%. This underrepresentation is consistent with data from the landmark EVEREST-II, COAPT, and Percutaneous Repair with the MitraClip Device for Severe Functional/Secondary Mitral Regurgitation (MITRA-FR) trials, in which the prevalence of enrolled females was 36.2%, 36.0%, and 25.3%, respectively [12,13,18]. Similarly, real-world TEER collective or registry datasets draw a comparable picture, at least for Europe. Two recently published meta-analyses report a prevalence of female patients of 38.6% and 45.6%, respectively [19,20]. It must be noted that the majority of the data in the latter meta-analysis by Ya’Qoub et al. are derived from two U.S. registries published by Villablanca and colleagues and Khan et al., which report a female prevalence of 47.6% and 47.0%, respectively [21,22], and thus obviously do not correspond to the care situation in Europe. If these two U.S. studies are excluded from the meta-analysis and only the European data are considered, the prevalence of women undergoing TEER is again only 37.6%. Together with the finding that women are significantly older than men at the timing of TEER, which has been repeatedly shown and is also confirmed by the present dataset [23,24,25], this may suggest that female patients are referred to percutaneous MV repair both less frequently and at later stages of the disease. Potential reasons for this perceived delay in treatment and undertreatment of women, which have been repeatedly reported and do not appear to be a specific problem in the treatment of MR [17], are still largely obscure and urgently require further exploration.

Regarding clinical characteristics at baseline, the present data indicate that women had fewer comorbidities, often less ischemic heart disease, reflected by a lower prevalence of coronary artery disease (CAD) or previous CABG surgery, which was also translated into a lower proportion of reduced left ventricular ejection fraction (LVEF) and a lower number of pre-existing implantable cardioverter/defibrillators (ICD) than in male patients. These findings agree with results from a sex-specific post hoc analysis of the COAPT trial, the multicentric German Transcatheter Mitral Valve Intervention (TRAMI), the European Registry for Transcatheter Repair in Secondary Mitral Regurgitation (euroSMR), the aforementioned U.S. registries, and several single-center studies [21,22,23,24,25,26]. Although we cannot provide detailed echocardiographic parameters for ventricular geometry, the evidence from these studies shows that the indexed ventricular or annular dimensions are significantly smaller in female patients, accounting for the observation that fewer clips were needed, which reduced procedure times compared with male patients, as has previously been reported by a number of other studies [23,25,26,27].

As a key finding, the present study shows that TEER was equally safe and effective in both sexes, as evidenced by comparable sex-specific MR reduction, procedure-related complications, and in-hospital mortality. There are partly inconsistent results reported in the literature for these relevant short-term outcome measures. Comparable safety and efficacy between sexes has been demonstrated in the highly selected patient populations of randomized controlled trials (RCTs), such as EVEREST-II, COAPT, and MITRA-FR, as well as in multicenter registry studies conducted across Europe, such as the two-phase observational study of the MitraClip system in Europe (ACCESS-EU) and the euroSMR registry [12,18,25,26,28]. In contrast, the German TRAMI registry reports a higher rate of major bleeding events in women, and analyses of the US National Inpatient Sample (NIS) database of more than 14,000 patients indicate that periprocedural strokes are more frequent in female patients [22,24]. Both results are also confirmed by two recent meta-analyses, comprising 18,459 and 24,905 patients, respectively, but also including the studies just mentioned [20,29]. However, consistent with the present results, no significant differences in in-hospital or 30-day mortality between the sexes were found in the meta-analyses, in the three mentioned RCTs, or in the registry studies from the United States or Europe [12,18,20,22,25,26,29,30,31].

Another major finding of the present study is the higher cumulative survival rate of women compared with men three years after the TEER procedure, both in the unadjusted data analysis and in the propensity score-matched analysis. In the same direction, a significant association between female sex and lower all-cause mortality was detected in the multivariate regression analysis. Overall, there are only a few studies addressing long-term sex-specific outcomes after TEER, with most of them reporting unadjusted survival rates with limited meaningfulness. However, restricting the framing of the present results to only the adjusted event rates published to date, our finding is consistent with the emerging signal that women have higher long-term survival rates than men after TEER. Thus, in the sample of more than 5000 TEER patients drawn from the US NIS database, Villablanca et al. reported an association between female sex and a lower one-year adjusted risk of all-cause mortality [21]. This is also confirmed in a recently published Japanese multicenter propensity score-matched study of patients with functional MR who underwent TEER, as well as in the meta-analysis by Ya’Qoub and colleagues mentioned above, which also adjusted for confounders [20,32]. However, a possible poorer long-term outcome of females after TEER [33], as repeatedly reported for surgically treated collectives [8,9,10], or no sex-specific difference, also cannot be completely excluded [19,25]; therefore, in addition to our study, further high-quality investigations would be desirable for a conclusive clarification. This also applies to the derivation of an explanation for the emerging finding of sex-specific disparities in adjusted mortality after TEER, which can only be speculated about in the light of the data available so far. According to recent studies, women possibly experience a more pronounced left ventricular reverse remodeling after TEER, which may account for their better long-term outcome [34,35]. Furthermore, this could be partly due to the more favorable comorbidity profile in females and possible unmeasured confounders at baseline, differences in the treatment adherence, or other unexplored differences in sex-specific biology.

Given the high prevalence of AF in the cohort of TEER patients, reflecting the complex and intimate pathophysiological interactions with MR, and its dismal impact on long-term prognosis, which has been confirmed in multiple additional studies [36,37,38,39,40], we felt prompted to perform a sex-specific analysis of long-term outcome, stratified by the status of concomitant AF. Here, we demonstrate for the first time that concomitant AF abolishes the prognostic advantage of females over males and, in contrast to males, significantly impairs long-term survival in women undergoing TEER. In contextualizing these findings, existing evidence gaps related to sex disparities also in AF become apparent. Thus, there is limited research addressing the sex-specific pathogenesis, management, and outcome, even in the general AF population. Regarding the outcome parameter long-term mortality, which is addressed in the present study, there is conflicting evidence on the effect of AF on the risk of death in females. However, the largest and most methodologically sound meta-analysis to date, including more than 4.3 million patients enrolled in 30 studies, correspondingly shows an association between AF and a higher risk of all-cause mortality in women compared with men [41]. Here, as in the present study, the reasons for the sex differences in mortality risk remain unclear. In particular, as we found no obvious differences in the underlying treatment of AF or the use of oral anticoagulants between the sexes in our cohort of TEER patients, the possible reasons for the sex-specific disparities in long-term mortality are not overt and can only be conjectured. As the use of antiarrhythmic drugs for rhythm control was not insignificant in the present collective, sex differences in the side-effect profile of the medications used may have played a potential role. Thus, it has been shown that women are at greater risk for ventricular arrhythmias, an often-fatal side effect of antiarrhythmics [42]. In addition, a higher risk of bleeding has been reported for women, which may be due to a differential response to oral anticoagulants [43]. Furthermore, as already mentioned, there is a possibility that unrecorded confounders could have biased the result. Future research needs to clarify the potential mechanisms that underlie the differential outcome effects of AF observed in our analysis and whether a specific sex-tailored treatment strategy for concomitant AF can enhance prognosis.

## 5. Limitations

Using established statistical methods of propensity score matching and multivariable Cox regression analysis, we aimed to minimize the effects of confounding factors; however, we cannot exclude the possibility of residual bias due to unknown variables that cannot be corrected for. In addition, some of the analyzed registries were managed retrospectively. Furthermore, functional data, including echocardiographic assessment of MR severity, as well as major cardiac and cerebrovascular events or the specific cause of death during follow-up, were not fully available for the entire cohort. Relative to clinical trials, the proportion of missing data, although small, and the use of a registry, may limit accuracy, which may reduce internal validity. Nevertheless, clinically highly relevant endpoints were addressed. 

## 6. Conclusions

With the present study, we provide an in-depth analysis of sex disparities in a large and well-characterized all-comer multicenter collective of patients undergoing TEER. In the population studied, female patients appear to be underrepresented relative to the burden of MR in the general population, and are older and less comorbid than male patients. Furthermore, the TEER procedure is equally safe and effective in both sexes. While in-hospital mortality did not differ, female patients experienced a significantly better adjusted long-term survival compared with male patients. Innovatively, we show that AF, the most common comorbid condition, offsets the prognostic advantage of females over males and, in contrast to males, significantly impairs long-term survival in women undergoing TEER. Further research is warranted to elucidate underlying causes of the observed sex disparities and to develop sex-tailored treatment recommendations resulting in more equitable health care. In this regard, addressing relevant comorbidities in TEER collectives, such as AF in particular, potentially represents an impactful approach for further prognostic improvements.

## Figures and Tables

**Table 1:** Clinical characteristics of the total cohort stratified by sex before and after propensity score matching at the time of the TEER procedure.

Variable	Overall (n = 821)	Before Propensity-Score-Matching			After Propensity-Score-Matching		
		Female (n = 307)	Male (n = 514)	*p*-Value	Female (n = 307)	Male (n = 307)	*p*-Value
Age (years)	78.3 ± 8	80.4 ± 6.7	77 ± 8.5	0.03	80.4 ± 6.6	79.8 ± 6.5	0.2
euroSCORE II *	16.0% (20)	13.1 (20.1)	16.5 (20.2)	0.054	13.1 (20.1)	14.9 (18)	0.9
STS Risk Score *	6.6% (8)	6.1 (7.5)	7 (8.6)	0.2	6.1 (7.5)	6.4 (7.1)	0.8
NYHA class I NYHA class II NYHA class III NYHA class IV	0.1% (1) 3.2% (26) 75.6% (621) 21.7% (173)	0.3% (1) 2.9% (9) 76.9% (236) 19.9% (61)	0% (0) 3.3% (17) 74.9% (385) 21.8% (112)	0.6	0.3% (1) 2.9% (9) 76.9% (236) 19.9% (61)	0% (0) 2.6% (8) 76.2% (234) 21.2% (65)	0.9
COPD	17.8% (146)	15.3% (47)	19.3% (99)	0.2	15.3% (47)	15.6% (48)	1
CAD	62.2% (511)	53.1% (163)	67.7% (348)	**<0.0001**	53.1% (163)	60.3% (185)	0.1
Prior CAB-OP	27.8% (228)	18.2% (56)	33.5% (172)	**<0.0001**	18.2% (56)	22.1% (68)	0.3
Prior PCI	54.0% (443)	50.5% (155)	56.0% (288)	0.1	50.5% (155)	54.1% (166)	0.4
Pre-existing ICD	22.3% (183)	9.4% (29)	30.0% (154)	**<0.0001**	9.4% (29)	11.1% (34)	0.6
Pre-existing CRT	14.4% (118)	7.2% (22)	18.7% (96)	**<0.0001**	7.2% (22)	10.1% (31)	0.3
Diabetes mellitus	29.8% (245)	28.7% (88)	30.5% (157)	0.6	28.7% (88)	27.7% (85)	0.9
Arterial hypertension	81% (665)	79.2% (243)	82.1% (422)	0.3	79.2% (243)	81.8% (251)	0.5
Prior Stroke	9.7% (80)	9.8% (30)	9.7% (50)	1	9.8% (30)	8.8% (27)	0.8
Atrial Fibrillation *Paroxysmal* *Non-paroxysmal*	74.1% (608) *21.2% (174*) *52.9% (434)*	70.7% (217) *21.8% (67)* *48.9% (150)*	76.1% (391) *20.8% (107)* *55.3% (284)*	0.1 *0.2* *0.2*	70.7% (217) *21.8% (67)* *48.9% (150)*	73.9% (227) *20.8% (64)* *53.1% (163)*	0.4 *0.2* *0.3*
LVEF ≥ 50% LVEF 41–49% LVEF ≤ 40%	39.2% (322) 11.6% (95) 49.2% (404)	50.5% (155) 13.4% (41) 36.2% (111)	32.5% (167) 10.5% (54) 57.0% (293)	**<0.0001**	50.5% (155) 13.4% (41) 36.2% (111)	46.9% (144) 13.4% (41) 39.7% (122)	0.6
GFR (mL/Min)	50 ± 25.5	48.5 ± 21.3	51.2 ± 27.7	0.1	48.5 ± 21	51.5 ± 31	0.2
NT-proBNP (ng/L) *	2262 (4936)	1919 (4328)	2463 (5288)	0.1	1919 (4328)	2465 (5028)	0.2
TR grade III	18.6% (153)	19.9% (61)	17.9% (92)	0.5	19.9% (61)	19.9% (61)	1
Degenerative MR etiology Functional MR etiology Mixed MR etiology	35.7% (293) 52.6% (432) 11.7% (96)	38.8% (119) 47.9% (147) 13.4% (41)	33.9% (174) 55.4% (285) 10.7% (55)	0.1	38.8% (119) 47.9% (147) 13.4% (41)	37.5% (115) 49.8% (153) 12.7% (39)	0.9
Heart Failure Therapy							
ACE-/AT1 Inhibitors	72.2% (593)	76.5% (235)	69.6% (358)	**0.04**	76.5% (235)	74.3% (228)	0.6
ARN Inhibitor	13.5% (111)	8.1% (25)	16.7% (86)	**0.004**	8.1% (25)	11.1% (34)	0.3
Beta Blockers	88.8% (729)	89.9% (276)	88.1% (453)	0.5	89.9% (276)	85.7% (263)	0.1
Loop diuretics	90.6% (744)	92.2% (283)	89.7% (461)	0.3	92.2% (283)	89.6% (275)	0.3
Thiazid diuretics	17.4% (143)	15.3% (47)	18.7% (96)	0.3	15.3% (47)	19.2% (59)	0.2
Aldosteron antagonists	48.2% (396)	45% (138)	50.2% (258)	0.1	45% (138)	45.3% (139)	1
Ivabradin	49.5% (10)	0.7% (2)	1.6% (8)	0.3	0.7% (2)	1% (3)	1
Digitalis	6.8% (56)	5.9% (18)	7.4% (38)	0.5	5.9% (18)	5.5% (17)	1
SGLT-II-Inhibitors	4.8% (39)	2.6% (8)	6.0% (31)	**0.02**	2.6% (8)	5.5% (17)	0.1
Vericiguat	0.1% (1)	0.0% (0)	0.2% (1)	1	0.0% (0)	0.0% (0)	1

Data presented as percentages or mean ± SD. * Data presented as median with interquartile range (IQR). AF—atrial fibrillation. COPD—chronic obstructive pulmonary disease. CABG—coronary artery bypass graft surgery. PCI—percutaneous coronary intervention. ICD—implantable cardioverter defibrillator. CRT—cardiac resynchronization therapy. GFR—glomerular filtration rate. LV function—left ventricular function. LA—left atrial. NT-proBNP—N-terminal pro-B-type natriuretic peptide. TR—tricuspid regurgitation. MR—mitral regurgitation. ACE—angiotensin converting enzyme. AT1—angiotensin II type 1 receptor. ARN—angiotensin receptor neprylisin. SGLT-II—sodium-glucose transporter 2. Statistically significant *p*-values are shown in bold. The italics are intended to indicate that this is a subcategory of the corresponding parameter (here AF).

**Table 2:** Procedural characteristics and efficacy outcomes of the total cohort stratified by sex before and after propensity score matching at the time of the TEER procedure.

Variable	Overall	Female	Male	*p*-Value
	(n = 821)	(n = 307)	(n = 514)	
Procedural duration (min) *	80 (60)	75 (54)	83 (63)	**0.003**
Number of clips implanted *	1 (1)	1 (1)	2 (1)	**<0.001**
Periprocedual MR reduction ^#^	Δ2.04 ± 0.6	Δ2 ± 0.6	Δ2.1 ± 0.6	0.1
Postprocedural MR grade ^#^				0.7
≤*mild-to-moderate*	88.6% (727)	88.9% (273)	88.3% (454)	
*moderate*	11.4% (94)	11.1% (34)	11.7% (60)	
Length of hospital stay (days) *	6 (5)	7 (4)	6 (5)	0.2

Data presented as percentages or mean ± SD. * Data presented as median with interquartile range (IQR). ^#^ MR grade according to American Society of Echocardiography (ASE) classification. Statistically significant *p*-values are shown in bold. The italics are intended to indicate that this is a subcategory of the corresponding parameter. The numbers should be in line with “≤mild-to-moderate” and “moderate”.

**Table 3:** Procedure-related complications and in-hospital mortality of the total cohort studied and stratified by sex before and after propensity score matching at the time of the TEER procedure.

Variable	Overall (n = 821)	Female (n = 307)	Male (n = 514)	*p*-Value
Stroke	0.6% (5)	0.3% (1)	0.8% (4)	0.7
Myocardial infarction	0% (0)	0% (0)	0% (0)	1
Bleeding complications *MVARC I* *MVARC II* *MVARC III* *MVARC IV* *MVARC V*	3.2% (26) *2.1% (17)* *0.6% (5)* *0.1% (1)* *0.4% (3)* *0% (0)*	3.3% (10) *1.6% (5)* *1.3% (4)* *0.3% (1)* *0% (0)* *0% (0)*	3.1% (16) *2.3% (12)* *0.2% (1)* *0% (0)* *0.6% (3)* *0% (0)*	1 *0.08*
Cardiac conduction system disturbances	0% (0)	0% (0)	0% (0)	1
In-hospital mortality *Cardiac cause* *Non-cardiac cause*	3.5% (29) *2.2% (18)* *1.3% (11)*	2.6% (8) *2% (6)* *0.6% (2)*	4.1% (21) *2.3% (12)* *1.8% (9)*	0.3 *0.8* *0.4*

Data presented as percentages and absolute numbers. MVARC—Mitral Valve Academy Research Consortium classification of bleeding events [16]. The italics are intended to indicate that this is a subcategory of the corresponding parameter.

**Table 4:** Clinical and procedural characteristics of males and females stratified by AF status.

	Male Sex			Female Sex		
Variable	No AF (n = 123)	AF (n = 391)	*p*-Value	No AF (n = 90)	AF (n = 217)	*p*-Value
Age (years)	75.0 ± 9.0	77.6 ± 8.3	**0.01**	79.0 ± 8	81.0 ± 6.0	0.1
euroSCORE II *	16.7 (18.5)	16.5 (21.4)	0.7	15.0 (17)	13.0 (21)	0.8
STS Risk Score *	6.9 (8.8)	7.0 (8.5)	0.4	5.5 (5.5)	6.7 (8.6)	**0.008**
NYHA class I NYHA class II NYHA class III NYHA class IV	0% (0) 2.4% (3) 78.9% (97) 18.7% (23)	0% (0) 3.6% (14) 73.7% (288) 22.8% (89)	0.6	0% (0) 6.7% (6) 75.6% (68) 17.8% (16)	0.5% (1) 1.4% (3) 77.4% (168) 20.7% (45)	0.08
COPD	21.1% (26)	18.7% (73)	0.6	18.9% (17)	13.8% (30)	0.3
CAD	73.2% (90)	66% (258)	0.2	58.9% (53)	50.7% (110)	0.2
Prior CAB-OP	42.3% (52)	30.7% (120)	**0.02**	22.2% (20)	16.6% (36)	0.3
Prior PCI	58.5% (72)	55.2% (216)	0.5	56.7% (51)	47.9% (104)	0.2
Pre-existing ICD	29.3% (36)	30.2% (118)	0.9	7.8% (7)	10.4% (22)	0.7
Pre-existing CRT	18.7% (23)	18.7% (73)	1	3.3% (3)	8.8% (19)	0.1
Diabetes mellitus	31.7% (39)	30.2% (118)	0.7	30% (27)	28.1% (61)	0.8
Arterial hypertension	81.3% (100)	82.4% (322)	0.8	68.9% (62)	83.4% (181)	**0.005**
Prior Stroke	6.5% (8)	10.7% (42)	0.2	8.9% (8)	10.1% (22)	0.8
LVEF ≥ 50% LVEF 41–49% LVEF ≤ 40%	25.2% (31) 8.9% (11) 65.9% (81)	34.8% (136) 11% (43) 54.2% (212)	0.08	45.6% (41) 10% (9) 44.4% (40)	52.5% (114) 14.7% (32) 32.7% (71)	0.2
GFR (mL/Min)	54 ± 26	51 ± 28	0.3	55 ± 23	46 ± 20	**0.03**
NT-proBNP (ng/L) *	2069 (6005)	2473 (5049)	0.6	2080 (4437)	1888 (3857)	0.6
TR grade III	11.4% (14)	19.9% (78)	**0.03**	10% (9)	24% (52)	**0.004**
Degenerative MR etiology Functional MR etiology Mixed MR etiology	35.8% (44) 57.7% (71) 6.5% (8)	33.2% (130) 54.7% (214) 12% (47)	0.2	35.6% (32) 52.2% (47) 12.2% (11)	40.1% (87) 46.1% (100) 13.8% (30)	0.6
Procedural duration (min) *	85 (62)	83 (63)	0.9	82 (39)	86 (49)	1
Number of clips implanted *	2 (1)	2 (1)	1	1 (1)	1 (1)	0.4
Periprocedual MR reduction ^#^	Δ2.0 ± 0.6	Δ2.1 ± 0.6	0.3	Δ1.9 ± 0.6	Δ2.0 ± 0.5	0.2
Length of hospital stay (days) *	6 (5)	6 (5)	0.6	6.5 (4)	7 (5)	0.1
Overall-MACCE *Cerebral/systemic thromboembolic event* *Bleeding requiring intervention* *In-hospital death from cardiovasc. cause*	6.5% (8) *0.8% (1)* *3.3% (4)* *2.4% (3)*	5.4% (21) *0.8% (3)* *3.1% (12)* *2.3% (9)*	0.7 *1* *1* *1*	6.7% (6) *0% (0)* *4.4% (4)* *2.2% (2)*	4.1% (9) *0.5% (1)* *2.8% (6)* *1.8% (4)*	0.4 *1* *0.5* *1*
In-hospital death from any cause	4.9% (6)	3.8% (15)	0.6	3.3% (3)	2.3% (5)	0.7
Heart Failure Therapy						
ACE/AT1 Inhibitors	66.7% (82)	70.6% (276)	0.4	80% (72)	75.1% (163)	0.4
ARN Inhibitor	17.1% (21)	16.6% (65)	0.9	7.8% (7)	8.3% (18)	1
Beta Blockers	87.8% (108)	88.2% (345)	0.9	87.8% (79)	90.8% (197)	0.4
Loop diuretics	86.2% (106)	90.8% (355)	0.2	90% (81)	93.1% (202)	0.4
Thiazid diuretics	18.7% (23)	18.7% (73)	1	13.3% (12)	16.1% (35)	0.6
Aldosteron antagonists	50.4% (62)	50.1% (196)	1	41.1% (37)	46.5% (101)	0.4
Ivabradin	4.1% (5)	0.8% (3)	**0.02**	1.1% (1)	0.5% (1)	0.5
Digitalis	0.8% (1)	9.5% (37)	**<0.001**	0% (0)	8.3% (18)	**0.002**
SGLT-II-Inhibitors	8.1% (10)	5.4% (21)	0.3	3.3% (3)	2.3% (5)	0.7
Vericiguat	0% (0)	0.3% (1)	1	0% (0)	0% (0)	1
Oral anticoagulants		**92.3% (361**)	-	**-**	**94% (204)**	**-**
VKA	-	45% (176)	-	-	44.7% (97)	-
NOAC	-	47.3% (185)	-	-	49.3% (107)	-

Data presented as percentages or mean ± SD. * Data presented as median with interquartile range (IQR). **^#^** MR grade according to American Society of Echocardiography (ASE) classification. AF—atrial fibrillation. COPD—chronic obstructive pulmonary disease. CABG—coronary artery bypass graft surgery. PCI—percutaneous coronary intervention. ICD—implantable cardioverter defibrillator. CRT—cardiac resynchronization therapy. GFR—glomerular filtration rate. LV function—left ventricular function. LA—left atrial. NT-proBNP—N-terminal pro-B-type natriuretic peptide. TR—tricuspid regurgitation. MR—mitral regurgitation. MACCE—major adverse cardiac and cerebrovascular events. AAD—antiarrhythmic drugs. ACE—angiotensin converting enzyme. AT1—angiotensin II type 1 receptor. ARN—angiotensin receptor neprylisin. SGLT-II—sodium-glucose transporter 2. VKA—vitamin k antagonist anticoagulants. NOAC—non-vitamin k antagonist oral anticoagulants. Statistically significant *p*-values are shown in bold.The italics are intended to indicate that this is a subcategory of the corresponding parameter.

## Data Availability

The data presented in this study is unavailable due to ethical restrictions.

## Supplementary Materials

The following supporting information can be downloaded at: https://www.mdpi.com/article/10.3390/jcm12237231/s1, Table S1: Comparison of clinical and procedural characteristics of patients lost to follow-up in comparison with those of patients not lost to follow-up; Table S2: Clinical and procedural characteristics of male patients with and without concomitant atrial fibrillation before and after propensity score matching; Table S3: Clinical and procedural characteristics of female patients with and without concomitant atrial fibrillation before and after propensity score matching; Table S4: Clinical and procedural characteristics of female and male patients with concomitant atrial fibrillation before and after propensity score matching; Table S5: Clinical and procedural characteristics of female and male patients without concomitant atrial fibrillation before and after propensity score matching.
