# Supplementary material for: Sex-Specific Disparities in Outcomes of Transcatheter Edge-to-Edge Repair for Mitral Regurgitation: A Multicenter “Real-World” Analysis
Source: J Clin Med. 2023 Nov 22;12(23):7231. doi: 10.3390/jcm12237231 (PMC10706895; doi:10.3390/jcm12237231)
Supplement: Supplementary file 1 [file jcm-12-07231-s001.zip › Supplemental_Table_S1.pdf]

Table S1: Comparison of clinical and procedural characteristics of patients lost to follow-up in comparison with those of patients not lost to follow-up. Data presented as percentages or mean ± SD. * - Data presented as median with interquartile range (IQR). # - MR grade according to American Society of Echocardiography (ASE) classification. AF – atrial fibrillation. COPD – chronic obstructive pulmonary disease. CABG – coronary artery bypass graft surgery. PCI – percutaneous coronary intervention. ICD – implantable cardioverter defibrillator. CRT – cardiac resynchronization therapy. GFR – glomerular filtration rate. LV function – left ventricular function. LA – left atrial. NT-proBNP – N-terminal pro-B-type natriuretic peptide. TR – tricuspid regurgitation. MR – mitral regurgitation. MACCE – major adverse cardiac and cerebrovascular events. AAD – antiarrhythmic drugs. ACE – angiotensin converting enzyme. AT1 – angiotensin II type 1 receptor. ARN – angiotensin receptor neprylisin. SGLT-II – sodium-glucose transporter 2.

Variable	Patients not lost to FU (n=766)	Patients lost to FU (n=55)	*p*-value
Age (years)	78 ± 8	79 ± 7	0.1
euroSCORE II*	16.1 (20.8)	12.4 (10.7)	0.3
STS Risk Score*	6.7 (8.4)	5.8 (5.2)	0.1
Male sex	61.9% (474)	72.7% (40)	0.1
NYHA class I<br>NYHA class II<br>NYHA class III<br>NYHA class IV	0.1% (1)<br>3.4% (26)<br>75.8% (581)<br>20.6% (158)	0% (0)<br>0% (0)<br>72.7% (40)<br>27.3% (15)	0.3
COPD	18.3% (140)	10.9% (6)	0.2
CAD	62.4% (478)	60% (33)	0.8
Prior CAB-OP	28.5% (218)	18.2% (10)	0.1
Prior PCI	53.9% (413)	54.5% (30)	1
Pre-existing ICD	22.5% (172)	20% (11)	0.7
Pacemaker<br>*CRT*	29.6% (227)<br>*14.4% (110)*	27.3% (15)<br>*14.5% (8)*	0.8<br>*1*
Diabetes mellitus	30.4% (233)	21.8% (12)	0.2
Arterial hypertension	81.5% (624)	74.5% (41)	0.2
Prior Stroke	9.4% (72)	14.5% (8)	0.2
LVEF ≥50%<br>LVEF 41-49%<br>LVEF ≤40%	38.8% (297)<br>11.4% (87)<br>49.9% (382)	45.5% (25)<br>14.5% (8)<br>40% (22)	0.3
Atrial fibrillation	73.9% (566)	76.4% (42)	0.8
GFR (mL/Min)	50 ± 26	53 ± 19	0.3
NT-proBNP (ng/L)*	2299 (4923)	2050 (5234)	0.6
AF patients after PVI	5.9% (45)	5.4% (3)	0.8
TR grade III	17.9% (137)	29.1% (16)	0.06
Degenerative MR etiology<br>Functional MR etiology<br>Mixed MR etiology	36.9% (278)<br>52.5% (402)<br>11.2% (86)	27.3% (15)<br>54.5% (30)<br>18.2% (10)	0.1
Median procedure duration (min)*	82 (62)	68 (34)	0.1
Number of clips implanted*	1 (1)	1 (0)	0.3
Periprocedual MR reduction#	Δ2.0 ± 0.6	Δ2.0 ± 0.3	0.6
Length of hospital stay (days)*	7 (5)	5 (4)	0.1
Overall-MACCE<br>*Cerebral/systemic thromboembolic event*<br>*Bleeding requiring intervention*<br>*In-hospital death from cardiovasc. cause*	5.5% (42)<br>*0.7% (5)*<br>*3.3% (25)*<br>*2.2% (17)*	3.6% (2)<br>*0% (0)*<br>*1.8% (1)*<br>*1.8% (1)*	0.8<br>*1*<br>*1*<br>*1*
In-hospital death from any cause	3.7% (28)	1.8% (1)	0.7
**Heart Failure Therapy**			
ACE-/AT1 Inhibitors	72.7% (557)	65.5% (36)	0.3
ARN Inhibitor	13.1% (100)	20% (11)	0.2
Beta Blockers	89.2% (683)	83.6% (46)	0.3
Diuretics	93.1% (713)	89.1% (49)	0.5
Aldosteron antagonists	48.4% (371)	45.5% (25)	0.7
SGLT-II-Inhibitors	4.6% (35)	7.3% (4)	0.5
Vericiguat	0% (0)	1.8% (1)	0.07
