# Supplementary material for: Sex-Specific Disparities in Outcomes of Transcatheter Edge-to-Edge Repair for Mitral Regurgitation: A Multicenter “Real-World” Analysis
Source: J Clin Med. 2023 Nov 22;12(23):7231. doi: 10.3390/jcm12237231 (PMC10706895; doi:10.3390/jcm12237231)
Supplement: Supplementary file 1 [file jcm-12-07231-s001.zip › Supplemental_Table_S2.pdf]

**Table S2: Clinical and procedural characteristics of male patients with and without concomitant atrial fibrillation before and after propensity score matching.** Data presented as percentages or mean ± SD. * - Data presented as median with interquartile range (IQR). # - MR grade according to American Society of Echocardiography (ASE) classification. AF – atrial fibrillation. COPD – chronic obstructive pulmonary disease. CABG – coronary artery bypass graft surgery. PCI – percutaneous coronary intervention. ICD – implantable cardioverter defibrillator. CRT – cardiac resynchronization therapy. GFR – glomerular filtration rate. LV function – left ventricular function. LA – left atrial. NT-proBNP – N-terminal pro-B-type natriuretic peptide. TR – tricuspid regurgitation. MR – mitral regurgitation. MACCE – major adverse cardiac and cerebrovascular events. AAD – antiarrhythmic drugs. ACE – angiotensin converting enzyme. AT1 – angiotensin II type 1 receptor. ARN – angiotensin receptor neprylisin. SGLT-II – sodium-glucose transporter 2.

| | | Before Propensity Score Matching | | | After Propensity Score Matching | | |
|---|---|---|---|---|---|---|---|
| Variable | Overall (n=514) | No AF (n=123) | AF (n=391) | *p*-value | No AF (n=123) | AF (n=246) | *p*-value |
| Age (years) | 77.0 ± 8.5 | 75.0 ± 9 | 77.6 ± 8.3 | **0.01** | 75.0 ± 9 | 76.0 ± 8 | 0.3 |
| euroSCORE II* | 16.5 (20.2) | 16.7 (18.5) | 16.5 (21.4) | 0.7 | 16.7 (18.5) | 18.3 (21.9) | 0.3 |
| STS Risk Score* | 7 (8.6) | 6.9 (8.8) | 7 (8.5) | 0.4 | 6.9 (8.8) | 7 (8.7) | 0.4 |
| NYHA class I<br>NYHA class II<br>NYHA class III<br>NYHA class IV | 0% (0)<br>3.3% (17)<br>74.9% (385)<br>21.8% (112) | 0% (0)<br>2.4% (3)<br>78.9% (97)<br>18.7% (23) | 0% (0)<br>3.6% (14)<br>73.7% (288)<br>22.8% (89) | 0.6 | 0% (0)<br>2.4% (3)<br>78.9% (97)<br>18.7% (23) | 0% (0)<br>2.4% (6)<br>71.5% (176)<br>26% (64) | 0.3 |
| COPD | 19.3% (99) | 21.1% (26) | 18.7% (73) | 0.6 | 21.1% (26) | 21.5% (53) | 1 |
| CAD | 67.7% (348) | 73.2% (90) | 66% (258) | 0.2 | 73.2% (90) | 74% (182) | 0.9 |
| Prior CAB-OP | 33.5% (172) | 42.3% (52) | 30.7% (120) | **0.02** | 42.3% (52) | 34.1% (84) | 0.1 |
| Prior PCI | 56% (288) | 58.5% (72) | 55.2% (216) | 0.5 | 58.5% (72) | 58.1% (143) | 1 |
| Pre-existing ICD | 30% (154) | 29.3% (36) | 30.2% (118) | 0.9 | 29.3% (36) | 35.8% (88) | 0.2 |

| | | | | | | | |
|---|---|---|---|---|---|---|---|
| Pre-existing CRT | 18.7% (96) | 18.7% (23) | 18.7% (73) | 1 | 18.7% (23) | 21.1% (52) | 0.7 |
| Diabetes mellitus | 30.5% (157) | 31.7% (39) | 30.2% (118) | 0.7 | 31.7% (39) | 32.1% (79) | 1 |
| Arterial hypertension | 82.1% (422) | 81.3% (100) | 82.4% (322) | 0.8 | 81.3% (100) | 81.7% (201) | 1 |
| Prior Stroke | 9.7% (50) | 6.5% (8) | 10.7% (42) | 0.2 | 6.5% (8) | 10.6% (26) | 0.3 |
| LVEF ≥50%<br>LVEF 41-49%<br>LVEF ≤40% | 32.5% (167)<br>10.5% (54)<br>57% (293) | 25.2% (31)<br>8.9% (11)<br>65.9% (81) | 34.8% (136)<br>11% (43)<br>54.2% (212) | 0.08 | 25.2% (31)<br>8.9% (11)<br>65.9% (81) | 27.6% (68)<br>11% (27)<br>61.4% (151) | 0.7 |
| GFR (mL/Min) | 51 ± 28 | 54 ± 26 | 51 ± 28 | 0.3 | 54 ± 26 | 54 ± 21 | 0.3 |
| NT-proBNP (ng/L)* | 2463 (5288) | 2069 (6005) | 2473 (5049) | 0.6 | 2069 (6005) | 2466 (5232) | 0.7 |
| TR grade III | 17.9% (92) | 11.4% (14) | 19.9% (78) | **0.03** | 11.4% (14) | 10.2% (25) | 0.7 |
| Degenerative MR etiology<br>Functional MR etiology<br>Mixed MR etiology | 33.9% (174)<br>55.4% (285)<br>10.7% (55) | 35.8% (44)<br>57.7% (71)<br>6.5% (8) | 33.2% (130)<br>54.7% (214)<br>12% (47) | 0.2 | 35.8% (44)<br>57.7% (71)<br>6.5% (8) | 30.9% (76)<br>60.6% (149)<br>8.5% (21) | 0.6 |
| Median procedure duration (min)* | 83 (63) | 85 (62) | 83 (63) | 0.9 | 85 (62) | 82 (63) | 0.9 |
| Number of clips implanted* | 2 (1) | 2 (1) | 2 (1) | 1 | 2 (1) | 2 (1) | 0.8 |
| Periprocedual MR reduction[#] | Δ2.1 ± 0.6 | Δ2.0 ± 0.6 | Δ2.1 ± 0.6 | 0.3 | Δ2.0 ± 0.6 | Δ2.1 ± 0.6 | 0.4 |
| Length of hospital stay (days)* | 6 (5) | 6 (5) | 6 (5) | 0.6 | 6 (5) | 6 (5) | 0.9 |
| Overall-MACCE<br>*Cerebral/systemic thromboembolic event* | 5.6% (29)<br>*0.8% (4)* | 6.5% (8)<br>*0.8% (1)* | 5.4% (21)<br>*0.8% (3)* | 0.7<br>*1* | 6.5% (8)<br>*0.8% (1)* | 5.7% (14)<br>*0.8% (1)* | 0.8<br>*1* |

| | | | | | | | |
|---|---|---|---|---|---|---|---|
| *Bleeding requiring intervention* | *3.1% (16)* | *3.3% (4)* | *3.1% (12)* | *1* | *3.3% (4)* | *3.3% (8)* | *1* |
| *In-hospital death from cardiovasc. cause* | *2.3% (12)* | *2.4% (3)* | *2.3% (9)* | *1* | *2.4% (3)* | *2.4% (6)* | *1* |
| In-hospital death from any cause | 4% (21) | 4.9% (6) | 3.8% (15) | 0.6 | 4.9% (6) | 4.5% (11) | 1 |
| **Heart Failure Therapy** | | | | | | | |
| ACE-/AT1 Inhibitors | 69.6% (358) | 66.7% (82) | 70.6% (276) | 0.4 | 66.7% (82) | 67.1% (165) | 1 |
| ARN Inhibitor | 16.7% (86) | 17.1% (21) | 16.6% (65) | 0.9 | 17.1% (21) | 19.9% (49) | 0.6 |
| Beta Blockers | 88.1% (453) | 87.8% (108) | 88.2% (345) | 0.9 | 87.8% (108) | 88.6% (218) | 0.9 |
| Loop diuretics | 89.7% (461) | 86.2% (106) | 90.8% (355) | 0.2 | 86.2% (106) | 89% (219) | 0.5 |
| Thiazid diuretics | 18.7% (96) | 18.7% (23) | 18.7% (73) | 1 | 18.7% (23) | 18.3% (45) | 1 |
| Aldosteron antagonists | 50.2% (258) | 50.4% (62) | 50.1% (196) | 1 | 50.4% (62) | 49.6% (122) | 0.9 |
| Ivabradin | 1.6% (8) | 4.1% (5) | 0.8% (3) | **0.02** | 4.1% (5) | 1.2% (3) | 0.1 |
| Digitalis | 7.4% (38) | 0.8% (1) | 9.5% (37) | **<0.001** | 0.8% (1) | 8.5% (21) | **0.002** |
| SGLT-II-Inhibitors | 6% (31) | 8.1% (10) | 5.4% (21) | 0.3 | 8.1% (10) | 4.1% (10) | 0.1 |
| Vericiguat | 0.2% (1) | 0% (0) | 0.3% (1) | 1 | 0% (0) | 0.4% (1) | 1 |
