# Supplementary material for: Sex-Specific Disparities in Outcomes of Transcatheter Edge-to-Edge Repair for Mitral Regurgitation: A Multicenter “Real-World” Analysis
Source: J Clin Med. 2023 Nov 22;12(23):7231. doi: 10.3390/jcm12237231 (PMC10706895; doi:10.3390/jcm12237231)
Supplement: Supplementary file 1 [file jcm-12-07231-s001.zip › Supplemental_Table_S3.pdf]

Table S3: Clinical and procedural characteristics of female patients with and without concomitant atrial fibrillation before and after propensity score matching. Data presented as percentages or mean ± SD. * - Data presented as median with interquartile range (IQR). # - MR grade according to American Society of Echocardiography (ASE) classification. AF – atrial fibrillation. COPD – chronic obstructive pulmonary disease. CABG – coronary artery bypass graft surgery. PCI – percutaneous coronary intervention. ICD – implantable cardioverter defibrillator. CRT – cardiac resynchronization therapy. GFR – glomerular filtration rate. LV function – left ventricular function. LA – left atrial. NT-proBNP – N-terminal pro-B-type natriuretic peptide. TR – tricuspid regurgitation. MR – mitral regurgitation. MACCE – major adverse cardiac and cerebrovascular events. AAD – antiarrhythmic drugs. ACE – angiotensin converting enzyme. AT1 – angiotensin II type 1 receptor. ARN – angiotensin receptor neprylisin. SGLT-II – sodium-glucose transporter 2.

	Before Propensity-Score-Matching				After Propensity-Score-Matching		
Variable	Overall (n=307)	No AF (n=90)	AF (n=217)	*p*-value	No AF (n=90)	AF (n=90)	*p*-value
Age (years)	80.0 ± 7	79.0 ± 8	81.0 ± 6	0.1	79.0 ± 8	81.0 ± 8	0.9
euroSCORE II*	13.1 (20.1)	15 (17)	13 (21)	0.8	15 (17)	13 (15.1)	0.7
STS Risk Score*	6.1 (7.5)	5.5 (5.5)	6.7 (8.6)	**0.008**	5.5 (5.5)	5.5 (4.7)	0.4
NYHA class I	0.3% (1)	0% (0)	0.5% (1)	0.08	0% (0)	1.1% (1)	0.2
NYHA class II	3% (9)	6.7% (6)	1.4% (3)		6.7% (6)	1.1% (1)	
NYHA class III	76.9% (236)	75.6% (68)	77.4% (168)		75.6% (68)	81.1% (73)	
NYHA class IV	19.9% (61)	17.8% (16)	20.7% (45)		17.8% (16)	16.7% (15)	
COPD	15.3% (47)	18.9% (17)	13.8% (30)	0.3	18.9% (17)	14.4% (13)	0.5
CAD	53.1% (163)	58.9% (53)	50.7% (110)	0.2	58.9% (53)	45.6% (41)	0.1
Prior CAB-OP	18.2% (56)	22.2% (20)	16.6% (36)	0.3	22.2% (20)	13.3% (12)	0.2
Prior PCI	50.5% (155)	56.7% (51)	47.9% (104)	0.2	56.7% (51)	44.4% (40)	0.1
Pre-existing ICD	9.4% (29)	7.8% (7)	10.4% (22)	0.7	7.8% (7)	10% (9)	0.8
Pre-existing CRT	7.2% (22)	3.3% (3)	8.8% (19)	0.1	3.3% (3)	10% (9)	0.1

Diabetes mellitus	28.7% (88)	30% (27)	28.1% (61)	0.8	30% (27)	20% (18)	0.2
Arterial hypertension	79.2% (243)	68.9% (62)	83.4% (181)	**0.005**	68.9% (62)	68.9% (62)	1
Prior Stroke	9.8% (30)	8.9% (8)	10.1% (22)	0.8	8.9% (8)	10% (9)	1
LVEF ≥50%	50.5% (155)	45.6% (41)	52.5% (114)		45.6% (41)	41.1% (37)	
LVEF 41-49%	13.4% (41)	10% (9)	14.7% (32)	0.2	10% (9)	26.7% (24)	0.1
LVEF ≤40%	36.2% (111)	44.4% (40)	32.7% (71)		44.4% (40)	32.2% (29)	
GFR (mL/Min)	49 ± 21	55 ± 23	46 ± 20	**0.03**	55 ± 23	49 ± 19	0.06
NT-proBNP (ng/L)*	1919 (4328)	2080 (4437)	1888 (3857)	0.6	2080 (4437)	2184 (4555)	0.7
TR grade III	20% (61)	10% (9)	24% (52)	**0.004**	10% (9)	10% (9)	1
Degenerative MR etiology	38.8% (119)	35.6% (32)	40.1% (87)		35.6% (32)	47.8% (43)	
Functional MR etiology	47.9% (147)	52.2% (47)	46.1% (100)	0.6	52.2% (47)	36.7% (33)	0.1
Mixed MR etiology	13.4% (41)	12.2% (11)	13.8% (30)		12.2% (11)	15.6% (14)	
Median procedure duration (min)*	75 (54)	82 (39)	86 (49)	1	82 (39)	78 (47)	0.9
Number of clips implanted*	1 (1)	1 (1)	1 (1)	0.4	1 (1)	1 (1)	0.8
Periprocedual MR reduction#	Δ2.0 ± 0.6	Δ1.9 ± 0.6	Δ2.0 ± 0.5	0.2	Δ1.9 ± 0.6	Δ2.1 ± 0.6	0.2
Length of hospital stay (days)*	7 (4)	6.5 (4)	7 (5)	0.1	6.5 (4)	6 (4)	0.2
Overall-MACCE	5.5% (17)	6.7% (6)	4.1% (9)	0.4	6.7% (6)	4.4% (4)	0.7
Cerebral/systemic thromboembolic event	*0.3% (1)*	*0% (0)*	*0.5% (1)*	*1*	*0% (0)*	*0% (0)*	*1*
Bleeding requiring intervention	*3.3% (10)*	*4.4% (4)*	*2.8% (6)*	*0.5*	*4.4% (4)*	*3.3% (3)*	*1*
In-hospital death from cardiovasc. cause	*2% (6)*	*2.2% (2)*	*1.8% (4)*	*1*	*2.2% (2)*	*1.1% (1)*	*1*
In-hospital death from any cause	2.6% (8)	3.3% (3)	2.3% (5)	0.7	3.3% (3)	3.3% (3)	1

**Heart Failure Therapy**							
ACE-/AT1 Inhibitors	76.5% (235)	80% (72)	75.1% (163)	0.4	80% (72)	76.7% (69)	0.7
ARN Inhibitor	8.1% (25)	7.8% (7)	8.3% (18)	1	7.8% (7)	10% (9)	0.8
Beta Blockers	89.9% (276)	87.8% (79)	90.8% (197)	0.4	87.8% (79)	92.2% (83)	0.5
Loop diuretics	92.2% (283)	90% (81)	93.1% (202)	0.4	90% (81)	88.9% (80)	1
Thiazid diuretics	15.3% (47)	13.3% (12)	16.1% (35)	0.6	13.3% (12)	15.6% (14)	0.8
Aldosteron antagonists	45% (138)	41.1% (37)	46.5% (101)	0.4	41.1% (37)	51.1% (46)	0.2
Ivabradin	0.6% (2)	1.1% (1)	0.5% (1)	0.5	1.1% (1)	0% (0)	1
Digitalis	5.9% (18)	0% (0)	8.3% (18)	**0.002**	0% (0)	5.6% (5)	0.06
SGLT-II-Inhibitors	2.6% (8)	3.3% (3)	2.3% (5)	0.7	3.3% (3)	1.1% (1)	0.6
Vericiguat	0% (0)	0% (0)	0% (0)	1	0% (0)	0% (0)	1
