# Supplementary material for: Sex-Specific Disparities in Outcomes of Transcatheter Edge-to-Edge Repair for Mitral Regurgitation: A Multicenter “Real-World” Analysis
Source: J Clin Med. 2023 Nov 22;12(23):7231. doi: 10.3390/jcm12237231 (PMC10706895; doi:10.3390/jcm12237231)
Supplement: Supplementary file 1 [file jcm-12-07231-s001.zip › Supplemental_Table_S4.pdf]

Table S4: Clinical and procedural characteristics of female and male patients with concomitant atrial fibrillation before and after propensity score matching. Data presented as percentages or mean ± SD. * - Data presented as median with interquartile range (IQR). # - MR grade according to American Society of Echocardiography (ASE) classification. AF – atrial fibrillation. COPD – chronic obstructive pulmonary disease. CABG – coronary artery bypass graft surgery. PCI – percutaneous coronary intervention. ICD – implantable cardioverter defibrillator. CRT – cardiac resynchronization therapy. GFR – glomerular filtration rate. LV function – left ventricular function. LA – left atrial. NT-proBNP – N-terminal pro-B-type natriuretic peptide. TR – tricuspid regurgitation. MR – mitral regurgitation. MACCE – major adverse cardiac and cerebrovascular events. AAD – antiarrhythmic drugs. ACE – angiotensin converting enzyme. AT1 – angiotensin II type 1 receptor. ARN – angiotensin receptor neprylisin. SGLT-II – sodium-glucose transporter 2.

		Before Propensity-Score-Matching			After Propensity-Score-Matching		
Variable	Overall (n=608)	Female (n=217)	Male (n=391)	*p*-value	Female (n=217)	Male (n=217)	*p*-value
Age (years)	78.8 ± 7.6	80.9 ± 5.8	77.6 ± 8.3	**<0.001**	80.9 ± 5.8	81 ± 5	0.7
euroSCORE II*	15.9 (21.2)	13 (21)	16.5 (21.4)	0.08	13 (21)	14 (19)	0.8
STS Risk Score*	7 (8.5)	6.7 (8.6)	7 (8.5)	0.8	6.7 (8.6)	6 (5.9)	0.2
NYHA class I	0.2% (1)	0.5% (1)	0% (0)	0.2	0.5% (1)	0% (0)	0.7
NYHA class II	2.8% (17)	1.4% (3)	3.6% (14)		1.4% (3)	2.8% (6)	
NYHA class III	75% (456)	77.4% (168)	73.7% (288)		77.4% (168)	76% (165)	
NYHA class IV	22% (134)	20.7% (45)	22.8% (89)		20.7% (45)	21.2% (46)	
COPD	16.9% (103)	13.8% (30)	18.7% (73)	0.1	13.8% (30)	14.7% (32)	0.9
CAD	60.5% (368)	50.7% (110)	66% (258)	**<0.001**	50.7% (110)	56.2% (122)	0.3
Prior CAB-OP	25.6% (156)	16.6% (36)	30.7% (120)	**<0.001**	16.6% (36)	18% (39)	0.8
Prior PCI	52.6% (320)	47.9% (104)	55.2% (216)	0.09	47.9% (104)	53% (115)	0.3
Pre-existing ICD	23% (140)	10.1% (22)	30.2% (118)	**<0.001**	10.1% (22)	12% (26)	0.6

Pre-existing CRT	15.1% (92)	8.8% (19)	18.7% (73)	**<0.001**	8.8% (19)	11.1% (24)	0.5
Diabetes mellitus	29.4% (179)	28.1% (61)	30.2% (118)	0.6	28.1% (61)	27.2% (59)	0.9
Arterial hypertension	82.7% (503)	83.4% (181)	82.4% (322)	0.8	83.4% (181)	82.9% (180)	1
Prior Stroke	10.5% (64)	10.1% (22)	10.7% (42)	0.9	10.1% (22)	9.2% (20)	0.9
LVEF ≥50%<br>LVEF 41-49%<br>LVEF ≤40%	46% (280)<br>12.3% (75)<br>46.5% (283)	52.5% (144)<br>14.7% (32)<br>32.7% (71)	34.8% (136)<br>11% (43)<br>54.2% (212)	**<0.001**	52.5% (144)<br>14.7% (32)<br>32.7% (71)	49.3% (107)<br>13.8% (30)<br>36.9% (80)	0.7
Paroxysmal AF<br>Persistent AF<br>Permanent AF	29.1% (177)<br>32.1% (195)<br>38.8% (236)	31.3% (68)<br>33.6% (73)<br>35% (76)	27.9% (109)<br>31.2% (122)<br>40.9% (160)	0.4	31.3% (68)<br>33.6% (73)<br>35% (76)	24.9% (54)<br>30.9% (67)<br>44.2% (96)	0.1
GFR (mL/Min)	49 ± 26	46 ± 20	51 ± 28	**0.02**	46 ± 20	50 ± 32	0.1
NT-proBNP (ng/L)*	2294 (4810)	1888 (3857)	2473 (5049)	0.1	1888 (3857)	2691 (4796)	0.1
TR grade III	21.4% (130)	24% (52)	19.9% (78)	0.3	24% (52)	20.7% (45)	0.5
Degenerative MR etiology<br>Functional MR etiology<br>Mixed MR etiology	35.7% (217)<br>51.4% (314)<br>12.7% (77)	40.1% (87)<br>46.1% (100)<br>13.8% (30)	33.2% (130)<br>54.7% (214)<br>12% (47)	0.1	40.1% (87)<br>46.1% (100)<br>13.8% (30)	36.4% (79)<br>47.9% (104)<br>15.7% (34)	0.7
Median procedure duration (min)*	80 (60)	74 (55)	83 (63)	**0.01**	74 (55)	80 (57)	0.2
Number of clips implanted*	1 (1)	1 (1)	2 (1)	**<0.001**	1 (1)	2 (1)	**<0.001**
Periprocedual MR reduction	Δ2 ± 0.6	Δ2 ± 0.5	Δ2 ± 0.6	0.3	Δ2 ± 0.5	Δ2 ± 0.6	0.4

(carpentier grade)							
Length of hospital stay (days)*	6 (5)	7 (5)	6 (5)	**0.03**	7 (5)	6 (5)	**0.03**
Overall-MACCE	4.9% (30)	4.1% (9)	5.4% (21)	0.6	4.1% (9)	6.5% (14)	0.4
Cerebral/systemic thromboembolic event	*0.6% (4)*	*0.5% (1)*	*0.8% (3)*	*1*	*0.5% (1)*	0.5% (1)	*1*
Bleeding requiring intervention	*2.9% (18)*	*2.8% (6)*	*3.1% (12)*	*1*	*2.8% (6)*	2.7% (8)	*0.8*
In-hospital death from cardiovasc. cause	*2.1% (13)*	*1.8% (4)*	*2.3% (9)*	*0.8*	*1.8% (4)*	3.2% (7)	*0.5*
In-hospital death from any cause	3.3% (20)	2.3% (5)	3.8% (15)	0.4	2.3% (5)	4.1% (9)	0.4
**Heart Failure Therapy**							
ACE-/AT1 Inhibitors	72% (439)	75.1% (163)	70.6% (276)	0.3	75.1% (163)	77.4% (168)	0.7
ARN Inhibitor	13.7% (83)	8.3% (18)	16.6% (65)	**0.004**	8.3% (18)	10.1% (22)	0.6
Beta Blockers	89.1% (542)	90.8% (197)	88.2% (345)	0.4	90.8% (197)	86.6% (188)	0.2
Loop diuretics	91.6% (557)	93.1% (202)	90.8% (355)	0.4	93.1% (202)	94% (204)	0.8
Thiazid diuretics	17.8% (108)	16.1% (35)	18.7% (73)	0.5	16.1% (35)	18% (39)	0.7
Aldosteron antagonists	48.8% (297)	46.5% (101)	50.1% (196)	0.4	46.5% (101)	44.7% (97)	0.8
Ivabradin	0.6% (4)	0.5% (1)	0.8% (3)	1	0.5% (1)	0.5% (1)	1
Digitalis	9% (55)	8.3% (18)	9.5% (37)	0.7	8.3% (18)	7.4% (16)	0.9
SGLT-II-Inhibitors	4.3% (26)	2.3% (5)	5.4% (21)	0.09	2.3% (5)	5.1% (11)	0.2
Vericiguat	0.2% (1)	0% (0)	0.3% (1)	1	0% (0)	0% (0)	1
**Atrial Fibrillation Therapy**							

**Rate Control**	**49.2% (299)**	**52.1% (113)**	**52.4% (186)**	**0.9**	**52.1% (113)**	**57.1% (124)**	**0.3**
Beta Blockers	47% (286)	47% (102)	46.5% (182)	0.7	47% (102)	48.8% (106)	1
Additional Pacemaker	8.1% (49)	7.4% (16)	8.4% (33)	0.8	7.4% (16)	8.8% (19)	0.7
Additional Digitalis	6.7% (41)	6.5% (14)	6.9% (27)	0.9	6.5% (14)	6.5% (1)	1
**Rhythm Control**	**47.7% (290)**	**47.9% (104)**	**47.6% (186)**	**0.9**	**47.9% (104)**	**42.9% (93)**	**0.3**
Class-I-AAD	0.3% (2)	0.9% (2)	0% (0)	0.1	0.9% (2)	0% (0)	0.5
Class-II-AAD	27.1% (165)	30.4% (66)	25.3% (99)	0.2	30.4% (66)	24.4% (53)	0.2
Class-III-AAD	2.3% (14)	3.2% (7)	1.8% (7)	0.3	3.2% (7)	2.8% (6)	1
Class-II + Class-III-AAD	14% (85)	11.5% (25)	15.3% (60)	0.2	11.5% (25)	12% (26)	1
Additional PVI	7.9% (48)	6.5% (14)	8.7% (34)	0.4	6.5% (14)	6.9% (15)	1
**Oral anticoagulants**							
VKA	45.1% (274)	45.2% (98)	45% (176)	1	45.2% (98)	42.9% (93)	0.7
NOAC	48% (292)	49.3% (107)	47.3% (185)	0.7	49.3% (107)	49.8% (108)	1
