# Supplementary material for: Sex-Specific Disparities in Outcomes of Transcatheter Edge-to-Edge Repair for Mitral Regurgitation: A Multicenter “Real-World” Analysis
Source: J Clin Med. 2023 Nov 22;12(23):7231. doi: 10.3390/jcm12237231 (PMC10706895; doi:10.3390/jcm12237231)
Supplement: Supplementary file 1 [file jcm-12-07231-s001.zip › Supplemental_Table_S5.pdf]

Table S5: Clinical and procedural characteristics of female and male patients without concomitant atrial fibrillation before and after propensity score matching. Data presented as percentages or mean ± SD. * - Data presented as median with interquartile range (IQR). # - MR grade according to American Society of Echocardiography (ASE) classification. AF – atrial fibrillation. COPD – chronic obstructive pulmonary disease. CABG – coronary artery bypass graft surgery. PCI – percutaneous coronary intervention. ICD – implantable cardioverter defibrillator. CRT – cardiac resynchronization therapy. GFR – glomerular filtration rate. LV function – left ventricular function. LA – left atrial. NT-proBNP – N-terminal pro-B-type natriuretic peptide. TR – tricuspid regurgitation. MR – mitral regurgitation. MACCE – major adverse cardiac and cerebrovascular events. AAD – antiarrhythmic drugs. ACE – angiotensin converting enzyme. AT1 – angiotensin II type 1 receptor. ARN – angiotensin receptor neprylisin. SGLT-II – sodium-glucose transporter 2.

		Before Propensity-Score-Matching			After Propensity-Score-Matching		
Variable	Overall (n=213)	Female (n=90)	Male (n=123)	*p*-value	Female (n=90)	Male (n=90)	*p*-value
Age (years)	77 ± 9	79 ± 8	75 ± 9	**<0.001**	79 ± 8	77 ± 8	0.06
euroSCORE II*	16 (18.1)	15 (17.1)	16.7 (18.5)	0.5	15 (17.1)	16 (19.6)	0.5
STS Risk Score*	5.9 (7.6)	5.5 (5.5)	6.9 (8.8)	0.06	5.5 (5.5)	6 (7.4)	0.4
NYHA class I	0% (0)	0% (0)	0% (0)	0.3	0% (0)	0% (0)	0.4
NYHA class II	4.2% (9)	6.7% (6)	2.4% (3)		6.7% (6)	2.2% (6)	
NYHA class III	77.5% (165)	75.6% (68)	78.9% (97)		75.6% (68)	80% (68)	
NYHA class IV	18% (39)	17.8% (16)	18.7% (23)		17.8% (16)	17.8% (16)	
COPD	20.2% (43)	18.9% (17)	21.1% (26)	0.7	18.9% (17)	18.9% (17)	1
CAD	67.1% (143)	58.9% (53)	73.2% (90)	**0.04**	58.9% (53)	66.7% (60)	0.4
Prior CAB-OP	33.8% (72)	22.2% (20)	42.3% (52)	**0.003**	22.2% (20)	32.2% (29)	0.2
Prior PCI	57.7% (123)	56.7% (51)	58.5% (72)	0.9	56.7% (51)	54.4% (49)	0.9
Pre-existing ICD	20.2% (43)	7.8% (7)	29.3% (36)	**<0.001**	7.8% (7)	11.1% (10)	0.6

Pre-existing CRT	12.2% (26)	3.3% (3)	18.7% (23)	**<0.001**	3.3% (3)	12.2% (11)	0.06
Diabetes mellitus	31% (66)	30% (27)	31.7% (39)	0.9	30% (27)	31.1% (28)	1
Arterial hypertension	76.1% (162)	68.9% (62)	81.3% (100)	**0.05**	68.9% (62)	81.1% (73)	0.08
Prior Stroke	7.4% (16)	8.9% (8)	6.5% (8)	0.6	8.9% (8)	6.7% (6)	0.8
LVEF ≥50%<br>LVEF 41-49%<br>LVEF ≤40%	33.8% (72)<br>9.4% (20)<br>56.8% (121)	45.6% (41)<br>10% (9)<br>44.4% (40)	25.2% (31)<br>8.9% (11)<br>65.9% (81)	**0.004**	45.6% (41)<br>10% (9)<br>44.4% (40)	32.2% (29)<br>11.1% (10)<br>56.7% (51)	0.2
GFR (mL/Min)	54 ± 25	54.9 ± 23	53.5 ± 26	0.7	54.9 ± 23	54 ± 26	0.8
NT-proBNP (ng/L)*	2069 (5727)	2080 (4437)	2069 (6005)	0.4	2080 (4437)	2068 (6807)	0.4
TR grade III	10.8% (23)	10% (9)	11.4% (14)	0.8	10% (9)	8.9% (8)	1
Degenerative MR etiology<br>Functional MR etiology<br>Mixed MR etiology	35.7% (76)<br>55.4% (118)<br>8.9% (19)	35.6% (32)<br>52.2% (47)<br>12.2% (11)	35.8% (44)<br>57.7% (71)<br>6.5% (8)	0.3	35.6% (32)<br>52.2% (47)<br>12.2% (11)	38.9% (35)<br>54.4% (49)<br>6.7% (6)	0.5
Median procedure duration (min)*	80 (60)	77.5 (46.5)	85 (61.5)	0.1	77.5 (46.5)	82 (63)	0.2
Number of clips implanted*	1 (1)	1 (1)	1 (2)	0.052	1 (1)	1.5 (1)	0.2
Periprocedual MR reduction[#]	Δ2.0 ± 0.6	Δ1.9 ± 0.6	Δ2.0 ± 0.6	0.3	Δ1.9 ± 0.6	Δ2.0 ± 0.6	0.4
Length of hospital stay (days)*	6 (4)	6.5 (4)	6 (5)	0.6	6.5 (4)	6 (5)	0.5
Overall-MACCE<br>*Cerebral/systemic thromboembolic event*	6.6% (14)<br>*0.5% (1)*	6.7% (6)<br>*0% (0)*	6.5% (8)<br>*0.8% (1)*	1<br>*1*	6.7% (6)<br>*0% (0)*	8.9% (8)<br>*1.1% (1)*	0.8<br>*1*

Bleeding requiring intervention	*1.9% (4)*	*4.4% (4)*	*3.3% (4)*	*0.7*	*4.4% (4)*	*4.4% (4)*	*1*
In-hospital death from cardiovasc. cause	*2.3% (5)*	*2.2% (2)*	*2.4% (3)*	*1*	*2.2% (2)*	*3.3% (3)*	*1*
In-hospital death from any cause	4.2% (9)	3.3% (3)	4.9% (6)	0.7	3.3% (3)	4.4% (4)	1
**Heart Failure Therapy**							
ACE-/AT1 Inhibitors	72.3% (154)	80% (72)	66.7% (82)	**0.04**	80% (72)	68.9% (62)	0.1
ARN Inhibitor	13.1% (28)	7.8% (7)	17.1% (21)	0.06	7.8% (7)	12.2% (11)	0.5
Beta Blockers	87.8% (187)	87.8% (79)	87.8% (108)	1	87.8% (79)	86.7% (78)	1
Loop diuretics	87.8% (187)	90% (81)	86.2% (106)	0.5	90% (81)	86.7% (78)	0.6
Thiazid diuretics	16.4% (35)	13.3% (12)	18.7% (23)	0.4	13.3% (12)	18.9% (17)	0.4
Aldosteron antagonists	46.5% (99)	41.1% (37)	50.4% (62)	0.2	41.1% (37)	50% (45)	0.3
Ivabradin	2.8% (6)	1.1% (1)	4.1% (5)	0.4	1.1% (1)	3.3% (3)	0.6
Digitalis	0.5% (1)	0% (0)	0.8% (1)	1	0% (0)	1.1% (1)	1
SGLT-II-Inhibitors	6.1% (13)	3.3% (3)	8.1% (10)	0.2	3.3% (3)	7.8% (7)	0.3
Vericiguat	0% (0)	0% (0)	0% (0)	1	0% (0)	0% (0)	1
